# Identifying Functional Transcription Factor Binding Sites in Yeast by Considering Their Positional Preference in the Promoters

**DOI:** 10.1371/journal.pone.0083791

**Published:** 2013-12-26

**Authors:** Fu-Jou Lai, Chia-Chun Chiu, Tzu-Hsien Yang, Yueh-Min Huang, Wei-Sheng Wu

**Affiliations:** 1 Department of Engineering Science, National Cheng Kung University, Tainan, Taiwan; 2 Department of Electrical Engineering, National Cheng Kung University, Tainan, Taiwan; Inserm U869, France

## Abstract

Transcription factor binding site (TFBS) identification plays an important role in deciphering gene regulatory codes. With comprehensive knowledge of TFBSs, one can understand molecular mechanisms of gene regulation. In the recent decades, various computational approaches have been proposed to predict TFBSs in the genome. The TFBS dataset of a TF generated by each algorithm is a ranked list of predicted TFBSs of that TF, where top ranked TFBSs are statistically significant ones. However, whether these statistically significant TFBSs are functional (i.e. biologically relevant) is still unknown. Here we develop a post-processor, called the functional propensity calculator (FPC), to assign a functional propensity to each TFBS in the existing computationally predicted TFBS datasets. It is known that functional TFBSs reveal strong positional preference towards the transcriptional start site (TSS). This motivates us to take TFBS position relative to the TSS as the key idea in building our FPC. Based on our calculated functional propensities, the TFBSs of a TF in the original TFBS dataset could be reordered, where top ranked TFBSs are now the ones with high functional propensities. To validate the biological significance of our results, we perform three published statistical tests to assess the enrichment of Gene Ontology (GO) terms, the enrichment of physical protein-protein interactions, and the tendency of being co-expressed. The top ranked TFBSs in our reordered TFBS dataset outperform the top ranked TFBSs in the original TFBS dataset, justifying the effectiveness of our post-processor in extracting functional TFBSs from the original TFBS dataset. More importantly, assigning functional propensities to putative TFBSs enables biologists to easily identify which TFBSs in the promoter of interest are likely to be biologically relevant and are good candidates to do further detailed experimental investigation. The FPC is implemented as a web tool at http://santiago.ee.ncku.edu.tw/FPC/.

## Introduction

Cells respond to internal or external stimuli by changing their gene expression [1], [2], a process that a gene is transcribed by the RNA polymerase into an mRNA to convey information for ribosomes to synthesize proteins. A transcription factor (TF) is a protein that binds to a specific DNA sequence, called TF binding site (TFBS), in the promoter of a gene to regulate (activating/repressing) its transcription rate. With comprehensive knowledge of TFBSs, one can understand the transcriptional regulation of gene expression. Accordingly, researchers committed to developing experimental or computational approaches to identify TFBSs.

Some examples of the experimental approaches were the works carried out by experimental biologists [3]–[5]. These approaches were considerably expensive [6]. On the other hand, based on the partial conservation property of TFBS nucleotide sequences, computational approaches were applied to the identification of TFBSs. The most well-known classical method used position weight matrices (PWMs), carrying the frequencies and the variability of four nucleotides at each position for a DNA sequence in a quantitative manner. This approach searched for consensus sequences in the PWM and assumed that each nucleotide interacts with the TF independently [6], [7]. Although various degrees of success were made in this computational approach, the use of PWM for TFBS identification was reported to have a high false positive rate [8], [9]. Beside the classical approach, alternatives were developed mainly based on the discovery of the weak correlation when taking into account di-nucleotide or tri-nucleotide in the analysis of the interaction between a TF and the regulatory sequences [10], [11]. There were also approaches of TFBS identification using statistical algorithms such as Gibbs sampling [12] and artificial intelligence algorithms such as neural networks [13]. The identification quality is rather comparable with that by using the PWM [6]. However, because TFBSs are typically short and degenerate, both classical and alternative approaches of TFBS identification are subject to over-prediction. For improvement, a number of techniques or factors are considered in the literature: (i) extracting maximal sequence from experimentally identified binding sites to improve motif models [14], (ii) supplementing motif models with more genomic attributes such as evolutionary conservation [14], and (iii) considering the similarity of the gene expression profiles of a TF and its target genes as extra information [15].

**Figure 1:**
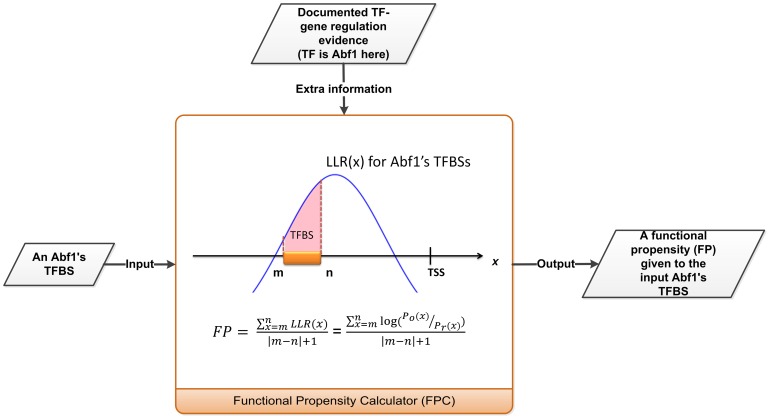
A conceptual diagram of the proposed functional propensity calculator (FPC) of Abf1's TFBSs. *Po(x)* and *Pr(x)* represent the observed and random distributions of Abf1's TFBS position relative to the TSS, respectively. Assume that an Abf1's TFBS is located from m bp to n bp relative to the TSS, then its functional propensity (FP) can be calculated as the formula shown in the figure.

Although the quality of TFBS identification is improved with the approaches mentioned above, whether or not the computationally predicted TFBSs are functional (i.e. biologically relevant) is still unknown. Therefore, for each TF, we develop a post-processor to evaluate the functionality of each TFBS of that TF in the existing computationally predicted TFBS datasets of that TF in the public domain. Our post-processor, called the functional propensity calculator (FPC), takes a TFBS as an input and outputs a functional propensity associated with the input TFBS. It is known that functional TFBSs reveal strong positional preference towards the transcriptional start site (TSS) [16]–[20]. This motivates us to take the TFBS position relative to the TSS as the key idea in building our FPC. A distinct FPC is constructed for the TFBSs of each TF. Namely, different FPCs are constructed for different TFs. Here we use the TF Abf1 as an example to illustrate how the FPC of Abf1's TFBSs works. First, high-confidence Abf1's TFBSs are extracted from the existing computationally predicted Abf1's TFBS dataset in the public domain. An Abf1's TFBS is called high-confidence if it is located in the promoter of a gene that is known to be regulated by Abf1 using the documented TF-gene regulation evidence in YEASTRACT database [21]. Second, an observed distribution of Abf1's TFBS position relative to the TSS is constructed using Abf1's high-confidence TFBSs. Third, a random distribution of Abf1's TFBS position relative to the TSS is constructed by assuming that the positions of Abf1's high-confidence TFBSs are uniformly distributed in the promoters. Finally, a functional propensity (FP) is given to each Abf1's TFBS based on the log likelihood ratio (LLR) of the observed and the random distributions at the position of that TFBS. Figure 1 exhibits the idea of our FPC using the TF Abf1 as an example (see Materials and Methods for details).

## Results

We reported the results (see Material S1 for details) of calculating functional propensities of the TFBSs in the existing computationally predicted TFBS datasets of 30 yeast TFs retrieved from MacIsaac et al.'s study [22], which used two well-known binding motif discovery algorithms, PhyloCon and Converge, to predict TFBSs. These 30 TFs were chosen because they all have at least 250 high-confidence TFBSs to construct the observed distribution of the TFBS position relative to the TSS. Our results are robust against different numbers of the required high-confidence TFBSs (see Discussion for details).

Note that the retrieved TFBS dataset of a TF is a ranked list of predicted TFBSs of that TF (sorted by the scores generated by the TFBS prediction algorithms in MacIsaac et al.'s study), where top ranked TFBSs are statistically significant ones. Here based on our calculated functional propensities, the TFBSs of a TF in the original TFBS dataset could be reordered, where top ranked TFBSs are now the ones with high functional propensities. To prove that our post-processor is effective in extracting functional TFBSs from the original TFBS dataset, we must show that the top ranked TFBSs in our reordered TFBS dataset are more likely to be functional (i.e. biologically relevant) than the top ranked TFBSs in the original TFBS dataset are. For this purpose, three published statistical tests (the functional enrichment test, the protein-protein interaction enrichment test and the expression coherence test) were performed.

Before introducing these three tests, let us define two terms. Let *Re(A,k)* and *Or(A,k)* be the set of genes whose promoters contain the “functional” TFBSs of TF *A*, where functional TFBSs of TF *A* are defined as the top *k*% of TF *A*'s TFBSs in our reordered and the original TFBS datasets, respectively. Note that *Re(A,k)* (or *Or(A,k)*) can be regarded as a set of genes that are co-regulated by TF *A* since their promoters all contain functional TFBSs of TF *A*.

### Functional enrichment test

Since genes whose promoters contain functional TFBSs of the same TF are likely to be co-regulated by that TF, they should perform similar molecular functions or be involved in similar biological processes [23]–[26]. Therefore, if the top ranked TFBSs in our reordered TFBS dataset are more functional (i.e. biologically relevant) than the top ranked TFBSs in the original TFBS dataset are, we expect that *Re(A,k)* is more enriched in the same Gene Ontology (GO) terms than *Or(A,k)* is. The functional enrichment test (proposed by Reimand et al. [25]) was used to check if our expectation is sustained. The procedure of this test was as follows. First, the GO Term Finder webserver [27] was used to find enriched GO terms in *Re(A,k)* and *Or(A,k)*, respectively. The GO terms were searched in all GO domains (biological process, molecular function and cellular component) and 0.05 was used as the false discovery rate (FDR) cut-off. Then a functional enrichment score (FES) was used to measure the enrichment of functional annotations in *Re(A,k)* and *Or(A,k)* by summing the absolute logarithms of the p-values of the enriched GO terms found in *Re(A,k)* and *Or(A,k)*, respectively.

**Figure 2:**
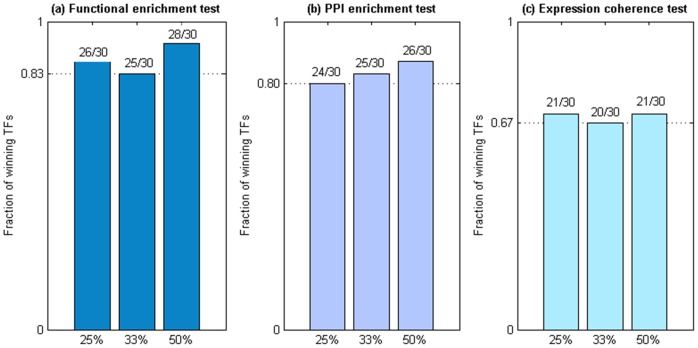
The outcomes of the three tests for the 30 TFs under study. *Re(A,k)* and *Or(A,k)* are the sets of genes whose promoters contain the “functional” TFBSs of TF A, where functional TFBSs of TF A are defined as the top k% of TF A's TFBSs in our reordered and the original TFBS datasets, respectively. For each of the 30 TFs under study, the three tests were performed on *Re(A,k)* and *Or(A,k)*, where k = 25, 33 or 50. The performance comparison results of (a) the functional enrichment test, (b) the PPI enrichment test, and (c) the expression coherence test are summarized. Note that TF A is called a winning TF if *Re(A,k)* outperforms *Or(A,k)* in the test and the fraction of the winning TFs is defined as the number of winning TFs divided by the total number of TFs under study. It can be seen that the fraction of the winning TFs is always greater than (a) 0.83 for the functional enrichment test, (b) 0.8 for the PPI enrichment test, and (c) 0.67 for the expression coherence test in all different scenarios, justifying the effectiveness of our post-processor in extracting functional TFBSs from the original TFBS dataset.

The functional enrichment test was applied to both *Re(A,k)* and *Or(A,k)* for each of the 30 TFs under study. Taking the TF Abf1 as an example, the functional enrichment test was applied to both *Re(Abf1,k)* and *Or(Abf1,k)*, where *k* = 25, 33 or 50. The same procedure was performed another 29 times for the other 29 TFs under study. As seen in Figure 2a, in most of the 30 TFs, *Re(A,k)* has a higher functional enrichment score than *Or(A,k)* does and this result is robust against different top *k*% (*k* = 25, 33 or 50) of the TFBSs used for defining functional TFBSs (see Material S2 for details). More precisely, *Re(A,k)* has a higher functional enrichment score than *Or(A,k)* does in 26 (25 or 28) out of the 30 TFs when *k* = 25 (33 or 50). In summary, the top ranked TFBSs in our reordered TFBS dataset display greater functional enrichment than the top ranked TFBSs in the original TFBS dataset do, justifying the effectiveness of our post-processor in extracting functional TFBSs from the original TFBS dataset.

### Protein-protein interaction enrichment test

It has been reported that co-regulated genes tend to physically interact with each other [28]. Since genes whose promoters contain functional TFBSs of the same TF are likely to be co-regulated by that TF, they should be enriched in physical protein-protein interactions (PPIs). Therefore, if the top ranked TFBSs in our reordered TFBS dataset are more functional (i.e. biologically relevant) than the top ranked TFBSs in the original TFBS dataset are, we expect that *Re(A,k)* is more enriched in physical PPIs than *Or(A,k)* is. The PPI enrichment test (proposed by Reimand et al. [25]) was used to check if our expectation is sustained. The procedure of this test was as follows. First, a PPI module associated with *Re(A,k)* was constructed. The physical PPI data were downloaded from the BioGRID database [29]. The genes that are in *Re(A,k)* and have physical PPIs with at least one gene in *Re(A,k)* are called core genes. The genes that are not in *Re(A,k)* but have physical PPIs with at least one core gene are called neighborhood genes. The core and neighborhood genes together formed the PPI module associated with *Re(A,k)*. Second, the statistical significance for the genes in *Re(A,k)* to be in the same PPI module was calculated using the hypergeometric distribution, which tests whether the ratio of genes in *Re(A,k)* to be in the same PPI module is significantly higher than random expectation.

The PPI enrichment test was applied to both *Re(A,k)* and *Or(A,k)* for each of the 30 TFs under study. The p-values calculated by the hypergeometric distribution were corrected to ensure FDR<0.05. As seen in Figure 2b, in most of the 30 TFs, *Re(A,k)* has a smaller p-value, i.e. a larger –log(p-value), than *Or(A,k)* does and this result is robust against different top *k*% (*k* = 25, 33 or 50) of the TFBSs used for defining functional TFBSs (see Material S2 for details). More precisely, *Re(A,k)* has a smaller p-value than Or(*A,k*) does in 24 (25 or 26) out of the 30 TFs when *k* = 25 (33 or 50). In summary, the top ranked TFBSs in our reordered TFBS dataset display higher tendency of having physical PPIs than the top ranked TFBSs in the original TFBS dataset do, justifying the effectiveness of our post-processor in extracting functional TFBSs from the original TFBS datasets.

### Expression coherence test

It has been reported that co-regulated genes tend to be co-expressed [23], [24]. Since genes whose promoters contain functional TFBSs of the same TF are likely to be co-regulated by that TF, they should have high tendency of being co-expressed. Therefore, if the top ranked TFBSs in our reordered TFBS dataset are more functional (i.e. biologically relevant) than the top ranked TFBSs in the original TFBS dataset are, we expect that *Re(A,k)* is more co-expressed than *Or(A,k)* is. The expression coherence test (proposed by Yang and Wu [26]) was used to check if our expectation is sustained. The expression data were downloaded from SPELL (Serial Pattern of Expression Levels Locator), which is a query-driven search engine for large gene expression microarray compendia [30]. The procedure of this test was as follows. First, two distributions were formed by computing the absolute value of the Pearson correlation coefficient between the expression data of any two genes in the *Re(A,k)* and *Or(A,k)*, respectively. Then one dataset is said to have higher expression coherence (i.e. more co-expressed) than the other if the mean of its distribution is stochastically greater than that of the other. The statistical significance was computed using Student's t-test.

The expression coherence test was applied to compare *Re(A,k)* and *Or(A,k)* for each of the 30 TFs under study. The p-values calculated by the Student's t-test were corrected to ensure FDR<0.05. As seen in Figure 2c, in most of the 30 TFs, *Re(A,k)* is more co-expressed (with corrected p-value <0.05) than *Or(A,k)* is and this result is robust against different top *k*% (*k* = 25, 33 or 50) of the TFBSs used for defining functional TFBSs (see Material S2 for details). More precisely, *Re(A,k)* is more co-expressed than *Or(A,k)* is in 21 (20 or 21) out of the 30 TFs when *k* = 25 (33 or 50). In summary, the top ranked TFBSs in our reordered TFBS dataset display higher tendency of being co-expressed than the top ranked TFBSs in the original TFBS dataset do, justifying the effectiveness of our post-processor in extracting functional TFBSs from the original TFBS dataset.

## Discussion

### Our result is better than random results

In the Results section, we have shown that the top ranked TFBSs selected by our functional propensity are more likely to be functional than are the top ranked TFBSs selected by the original TFBS prediction algorithm in MacIsaac et al.'s study. It would be more convincing if we can also show that the top ranked TFBSs selected by our functional propensity are more likely to be functional than are the top ranked TFBSs selected by random. Let *Re(A,k)* and *Ran(A,k)* be the set of genes whose promoters contain the “functional” TFBSs of TF *A*, where functional TFBSs of TF *A* are defined as the largest *k*% of TF *A*'s TFBSs selected by our functional propensity and by random. We generated 10 different *Ran(A,k)*s and performed the three tests (the functional enrichment test, the PPI enrichment test and the expression coherence test) on *Re(A,k)* and these 10 different *Ran(A,k)*s. As shown in Material S3, in almost all of the 30 TFs, *Re(A,k)* outperforms these 10 different *Ran(A,k)*s, suggesting that our result is of statistical significance.

### Our result is robust against different numbers of the required high-confidence TFBSs

In the Results section, the analyses of the TFBSs of 30 yeast TFs were reported. These 30 TFs were chosen because they all have at least 250 high-confidence TFBSs to construct the observed distribution of the TFBS location relative to the TSS. Changing the minimal number of high-confidence TFBSs that are required to construct the observed distribution would change the number of TFs that could be studied. For example, 22 (25, 27, 30, 44, 52 or 65) TFs can be studied if 400 (350, 300, 250, 200, 150 or 100) high-confidence TFBSs are required. Material S4 shows that our result is robust against different numbers of the required high-confidence TFBSs.

### Our result is robust against different existing computationally predicted TFBS datasets

In the Results section, the computationally predicted TFBS datasets were downloaded from MacIsaac et al.'s study. Here we wanted to check if our post-processor could work well for other TFBS datasets in the public domain. Therefore, we applied our post-processor to the computationally predicted TFBS datasets downloaded from SwissRegulon database [31]. SwissRegulon database deposited high-quality TFBS datasets predicted using Bayesian probabilistic analysis of a combination of input information including multiple alignments of orthologous inter-genic regions from related genomes and ChIP-chip binding data. Note that the retrieved TFBS dataset of a TF is a ranked list of predicted TFBSs of that TF (sorted by the scores generated by the TFBS prediction algorithm in SwissRegulon database), where top ranked TFBSs are statistically significant ones. Here based on our calculated functional propensities, the TFBSs of a TF in the original TFBS dataset could be reordered, where top ranked TFBSs are now the ones with high functional propensities. To prove that our post-processor is effective in extracting functional TFBSs from the original TFBS dataset, we must show that the top ranked TFBSs in our reordered TFBS dataset are more likely to be functional (i.e. biologically relevant) than the top ranked TFBSs in the original TFBS dataset are. For this purpose, three published statistical tests (the functional enrichment test, the PPI enrichment test and the expression coherence test) were performed. By requiring that a TF must have at least 400 high-confidence TFBSs to construct the observed distribution of the TFBS position relative to the TSS, the TFBS datasets of 20 yeast TFs in SwissRegulon database could be studied. As shown in Material S5, in most of the 20 TFs, the top ranked TFBSs in our reordered TFBS dataset outperform the top ranked TFBSs in the original TFBS dataset across all three tests, indicating that our result is robust against different computationally predicted TFBS datasets.

### Our result outperforms the original result in the ROC analysis

In order to check whether our reordered TFBS dataset (after applying our FPC) outperforms the original TFBS dataset in terms of the true positive rate and the false positive rate, the ROC (receiver operating characteristic) analysis was conducted. Before doing the ROC analysis, the benchmark set (i.e. the set of known functional TFBSs) has to be prepared. Since there is no such benchmark set available in the public domain, we instead prepared the benchmark set as follows. The prepared benchmark set consists of a TF's TFBSs which are located in the promoters of the genes that are known to be regulated by that TF using the literature evidence in YEASTRACT database [21]. The ROC analysis was applied to MacIsaac et al.'s TFBS datasets of 30 TFs (predicted by PhyloCon and Converge algorithms [22]) and the SwissRegulon's TFBS datasets of 20 TFs (predicted by MotEvo algorithm [31]). In almost all of the TFs under study, our reordered dataset has a larger AUC (area under curve) than the original dataset does and this result is robust against different TFBS prediction algorithms used (see Material S6 for details), justifying the effectiveness of our post-processor in extracting functional TFBSs from the original TFBS dataset.

### Our post-processor is useful for gene regulation study

**Figure 3:**
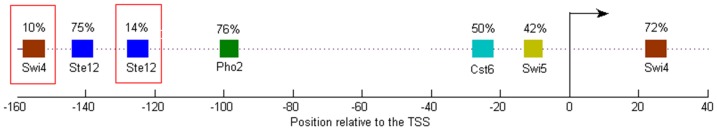
Seven putative TFBSs were predicted in the promoter of *FZF1*. In the promoter of *FZF1*, there are 7 computationally predicted TFBSs belonging to 5 different TFs (Swi4, Ste12, Pho2, Cst6, and Swi6) according to MacIsaac et al.'s TFBS datasets. Among these 7 putative TFBSs, our post-processor identified two TFBSs with high functional propensities to be worthy of further study. One is Swi4's TFBS, located from −158 bp to −152 bp relative to the TSS, whose functional propensity is within the largest 10% of the functional propensities of all computationally predicted Swi4's TFBSs, and the other one is Ste12's TFBS, located from −128 bp to −122 bp relative to the TSS, whose functional propensity is within the largest 14% of the functional propensities of all computationally predicted Ste12's TFBSs.

By analyzing MacIsaac et al.'s computationally predicted TFBS datasets, we found that 92.1% (6053/6576) of genes in the yeast genome have more than 7 predicted TFBSs in their promoters. These TFBSs could be recognized either by different TFs or by the same TF but located in different region of the promoter (see Figure 3 for an example). When dealing with a huge number of putative TFBSs in a promoter, biologists have troubles in determining which TFBSs should be chosen for further detailed experimental investigation. Our post-processor solved this problem by assigning functional propensities to TFBSs. The TFBSs with high functional propensities are likely to be biologically meaningful and are good candidates for further investigation. For example, in the promoter of *F* *Z* *F1*, there are 7 computationally predicted TFBSs belonging to 5 different TFs (Swi4, Ste12, Cst6, Pho2 and Swi6) according to MacIsaac et al.'s TFBS datasets (see Figure 3). Among these 7 TFBSs, our post-processor identified two TFBSs with high functional propensities to be worthy of further study. One is Swi4's TFBS, located from −158 bp to −152 bp relative to the TSS, whose functional propensity is within the largest 10% of the functional propensities of all computationally predicted Swi4's TFBSs, and the other one is Ste12's TFBS, located from −128 bp to −122 bp relative to the TSS, whose functional propensity is within the largest 14% of the functional propensities of all computationally predicted Ste12's TFBSs. Indeed, both TFs Swi4 and Ste12 are shown in literature to bind *F* *Z* *F1* in vivo [32], [33]. On the contrary, our post-processor predicted that the TFBSs of Cst6, Pho2 and Swi6 in the promoter of *F* *Z* *F1* may not be functional. Two kinds of evidence support our predictions. First, we cannot find any literature showing that Cst6, Pho2 and Swi6 can bind *F* *Z* *F1* in vivo. Second, Harbison et al.'s ChIP-chip data [34] show that Cst6, Pho2 and Swi6 indeed do not bind *F* *Z* *F1* in vivo for yeast cells cultured in the YPD medium. The p-values for Cst6-*F* *Z* *F1* binding, Pho2-*F* *Z* *F1* binding, and Swi6-*F* *Z* *F1* binding are all larger than 0.1, indicating that Cst6, Pho2 and Swi6 are very unlikely to bind *F* *Z* *F1* in vivo.

### Not every computationally predicted TFBS can be assigned a functional propensity by our method

Our method uses the relative distance of a TFBS to the TSS when computing its functional propensity. If a TFBS is located in the promoter of a gene whose TSS position is unknown, then our method cannot assign a functional propensity to the TFBS. Unfortunately, only the genomic coordinates of the TSSs of 4560 yeast genes are available from Nagalakshmi et al.'s work [35], which generated a high-resolution transcriptome of the yeast genome using a high-throughput RNA-seq method. Therefore, not every computationally predicted TFBS in the existing TFBS datasets can be assigned a functional propensity by our method. This problem can be solved in two ways. First, if we change our method to use the relative distance of a TFBS to the start codon when computing its functional propensity, then the problem is solved. The genomic coordinates of the start codons of all yeast genes are available from Saccharomyces Genome Database (SGD) [36]. However, we do not like this solution. We think that using the start codon as the reference is not biologically meaningful because the transcription process starts from the TSS but not from the start codon. The second way to solve this problem is waiting for the TSS information of the whole yeast genome released. With the rapid development of the high-throughput experimental technology, the genomic coordinates of the TSSs of all yeast genes should be available in the near future.

## Materials and Methods

### Data sources

Three data sources were used in this study. First, the genomic coordinates of the start codons, stop codons and the TSSs of 4560 genes were retrieved from Nagalakshmi et al.'s work [35], which generated a high-resolution transcriptome of the yeast genome using a high-throughput RNA-seq method. Second, the genomic coordinates of 409,513 TFBSs of 122 yeast TFs were retrieved from MacIsaac et al.'s work [22], which used two well-known binding motif discovery algorithms, PhyloCon and Converge, to predict TFBSs and generate the position weight matrices (PWMs). Note that the retrieved TFBS dataset of a TF is a ranked list (sorted by the PWM values) of the predicted TFBSs of that TF, where top ranked TFBSs are statistically significant ones. Third, the documented regulation evidence of 48,082 TF-gene pairs in yeast was downloaded from the YEASTRACT database [21]. The documented regulation evidence of a TF-gene pair comes from the literature showing at least one of the following two types of experimental evidence: (i) in vivo binding of the TF to the promoter of the gene and (ii) change in the expression of the gene due to the deletion (or mutation) of the TF-encoding gene.

### Construction of a distinct FPC for the TFBSs of each TF under study

A distinct FPC is constructed for the TFBSs of each TF. Namely, different FPCs are constructed for different TFs. Here we used the TF Abf1 as an example to illustrate how to construct the functional propensity calculator (FPC) for Abf1's TFBSs. First, from 760 computationally predicted Abf1's TFBSs in MacIsaac et al.'s study, 397 high-confidence Abf1's TFBSs were extracted. An Abf1's TFBS is called high-confidence if it is located in the promoter of a gene that is known to be regulated by Abf1 supported by the documented TF-gene regulation evidence in YEASTRACT database. Following previous studies [22], [32], [34], [37]–[39], the promoter of a gene is defined as the intergenic region between this gene and its nearest non-overlapped upstream gene. Second, an observed distribution of Abf1's TFBS position relative to the TSS was constructed using the 397 Abf1's high-confidence TFBSs. The construction process was as follows. Let *G* be the set of genes whose promoters contain at least one of the 397 high-confidence Abf1's TFBSs. Let *x* be a site relative to the TSS. For each *x* in the promoter of a gene in *G*, whether it is covered by any high-confidence Abf1's TFBSs was checked. The same process was applied to all genes in *G*. The total number, denoted as *Do(x)*, of Abf1's high-confidence TFBSs located at site *x* for all genes in *G* can be calculated. Then *Do(x)* is normalized to 1 and denoted as *Po(x)*. The *Po(x)* represents an estimation of the probability of observing an Abf1's TFBS at site *x*. Third, the random distribution of Abf1's TFBS location relative to the TSS, denoted as *Pr(x)*, was constructed by assuming that Abf1's high-confidence TFBSs are uniformly distributed in the promoters. The *Pr(x)* represents the probability of an Abf1's TFBS located at site *x* by random expectation. Finally, the functional propensity calculator (FPC) of Abf1's TFBSs was defined as the log likelihood ratio (LLR) of the observed distribution and the random distribution. That is, 
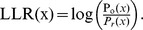


### Calculation of the functional propensity for each computationally predicted TFBS of a TF using the FPC

The functional propensity (FP) of a TFBS is defined as the average of the log likelihood ratios (LLRs) of the sites covered by the TFBS. Here we use the TF Abf1 as an example to illustrate how to calculate functional propensity for each computationally predicted Abf1's TFBS. If a computationally predicted Abf1's TFBS is located from m bp to n bp relative to the TSS, then its functional propensity (FP) can be calculated as 
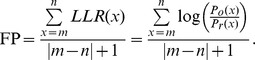



Using this formula, each of the computationally predicted Abf1's TFBSs in MacIsaac et al.'s TFBS dataset can be assigned a functional propensity. Finally, based on the functional propensities, these Abf1's TFBSs can be sorted from the highest to the lowest propensity. TFBSs which are at the top of the ranked list are likely to be functional ones.

## Supporting Information

Material S1 **Supplementary material 1 provides the functional propensities of the TFBSs in the existing computationally predicted TFBS datasets of 30 yeast TFs retrieved from MacIsaac et al.'s study.** The TFBSs of each TF are ranked from the largest to the smallest functional propensity. TFBSs which are at the top of the ranked list are likely to be functional ones.
(XLS)

Material S2 **Supplementary material 2 provides the detailed outcomes of the three tests (the functional enrichment test, the PPI enrichment test, and the expression coherence test) on** ***Re(A,k)*** **and** ***Or(A,k)*** **for the 30 TFs under study using the TFBS datasets retrieved from MacIsaac et al.'s study.**
(XLS)

Material S3 **Supplementary material 3 summarizes the outcomes of the three tests (the functional enrichment test, the PPI enrichment test, and the expression coherence test) on** ***Re(A,k)*** **and 10 different** ***Ran(A,k)*** **s for the 30 TFs under study using the TFBS datasets retrieved from MacIsaac et al.'s study.**
(PDF)

Material S4 **Supplementary material 4 summarizes the outcomes of the three tests (the functional enrichment test, the PPI enrichment test, and the expression coherence test) on our results requiring different numbers of the high-confidence TFBSs.**
(PDF)

Material S5 **Supplementary material 5 summarizes the outcomes of the three tests (the functional enrichment test, the PPI enrichment test, and the expression coherence test) on** ***Re(A,k)*** **and** ***Or(A,k)*** **for the 20 TFs under study using the TFBS datasets retrieved from SwissRegulon database.**
(PDF)

Material S6 **Supplementary material 6 provides the ROC analysis results of the TFBS datasets of 30 TFs retrieved from MacIsaac et al.'s study and the TFBS datasets of 20 TFs retrieved from SwissRegulon database.**
(PDF)
