# Supplementary material for: Identifying Functional Transcription Factor Binding Sites in Yeast by Considering Their Positional Preference in the Promoters
Source: PLoS One. 2013 Dec 26;8(12):e83791. doi: 10.1371/journal.pone.0083791 (PMC3873331; doi:10.1371/journal.pone.0083791)
Supplement: Material S3 — Supplementary material 3 summarizes the outcomes of the three tests (the functional enrichment test, the PPI enrichment test, and the expression coherence test) on Re(A,k) and 10 different Ran(A,k) s for the 30 TFs under study using the TFBS datasets retrieved from MacIsaac et al.'s study. (PDF) [file pone.0083791.s003.pdf]

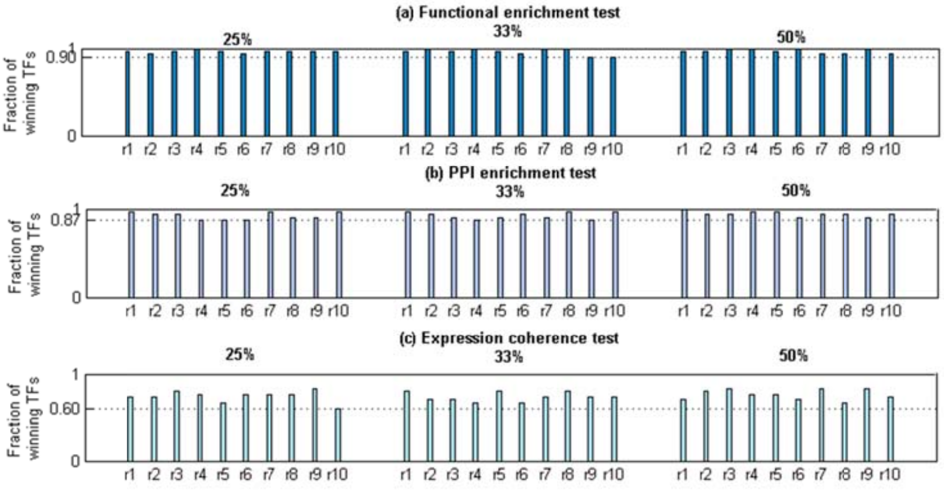

Supplementary Figure 1. The outcomes of the three tests on our results and 10 random results.

Re(A,k) and *Ran(A,k)* are the sets of genes whose promoters contain the "functional" TFBSs of TF *A*, where functional TFBSs of TF *A* are defined as the largest *k*% (*k*=25, 33, or 50) of TF *A*'s TFBSs selected by our functional propensity and by random, respectively. For each of the 30 TFs under study, the three tests were performed on *Re(A,k)* and these 10 different *Ran(A,k)*s, where *k*=25, 33 or 50. The performance comparison results of (a) the functional enrichment test, (b) the PPI enrichment test, and (c) the expression coherence test are summarized. Note that TF *A* is called a winning TF if *Re(A,k)* outperformed *Ran(A,k)* in the test and the fraction of winning TFs is defined as the number of winning TFs divided by the total number of TFs under study. It can be seen that the fraction of winning TFs is always greater than (a) 0.9 for the functional enrichment test, (b) 0.87 for the PPI enrichment test, and (c) 0.6 for the expression coherence test in all different scenarios, suggesting that our result is of statistical significance.
