# Supplementary material for: Identifying Functional Transcription Factor Binding Sites in Yeast by Considering Their Positional Preference in the Promoters
Source: PLoS One. 2013 Dec 26;8(12):e83791. doi: 10.1371/journal.pone.0083791 (PMC3873331; doi:10.1371/journal.pone.0083791)
Supplement: Material S4 — Supplementary material 4 summarizes the outcomes of the three tests (the functional enrichment test, the PPI enrichment test, and the expression coherence test) on our results requiring different numbers of the high-confidence TFBSs. (PDF) [file pone.0083791.s004.pdf]

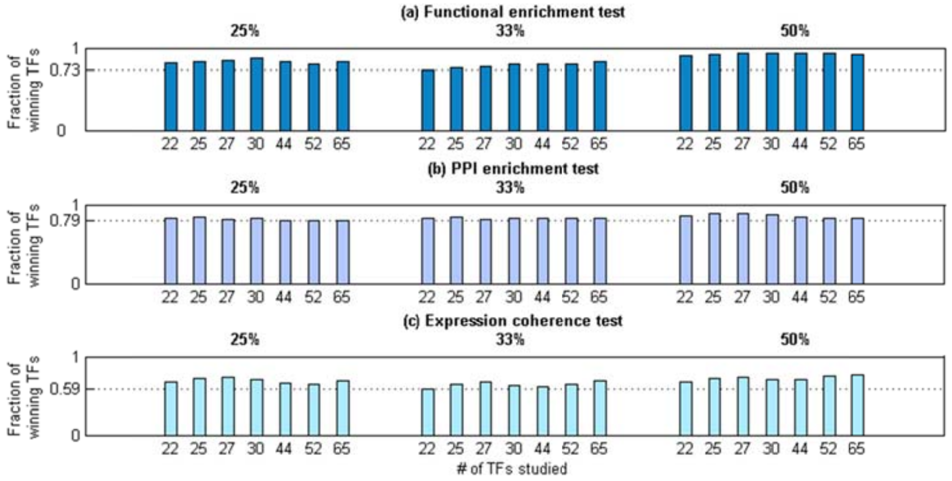

Supplementary Figure 2. The outcomes of the three tests on our results requiring different numbers of the high-confidence TFBSs.

Changing the minimal number of high-confidence TFBSs that are required to construct the observed distribution of TFBS position relative to the TSS would change the number of TFs that could be studied. For example, 22 (25, 27, 30, 44, 52 or 65) TFs can be studied if 400 (350, 300, 250, 200, 150 or 100) high-confidence TFBSs are required. For each of the TFs that can be studied, the three tests were performed on *Re(A,k)* and *Or(A,k)*, where *k*=25, 33 or 50. The performance comparison results of (a) the functional enrichment test, (b) the PPI enrichment test, and (c) the expression coherence test are summarized. Note that TF *A* is called a winning TF if *Re(A,k)* outperformed *Or(A,k)* in the test and the fraction of winning TFs is defined as the number of winning TFs divided by the total number of TFs under study. It can be seen that the fraction of winning TFs is always greater than (a) 0.73 for the functional enrichment test, (b) 0.79 for the PPI enrichment test, and (c) 0.59 for the expression coherence test in all different scenarios, proving that our result is robust against different numbers of the required high-confidence TFBSs.
