# Supplementary material for: Identifying Functional Transcription Factor Binding Sites in Yeast by Considering Their Positional Preference in the Promoters
Source: PLoS One. 2013 Dec 26;8(12):e83791. doi: 10.1371/journal.pone.0083791 (PMC3873331; doi:10.1371/journal.pone.0083791)
Supplement: Material S5 — Supplementary material 5 summarizes the outcomes of the three tests (the functional enrichment test, the PPI enrichment test, and the expression coherence test) on Re(A,k) and Or(A,k) for the 20 TFs under study using the TFBS datasets retrieved from SwissRegulon database. (PDF) [file pone.0083791.s005.pdf]

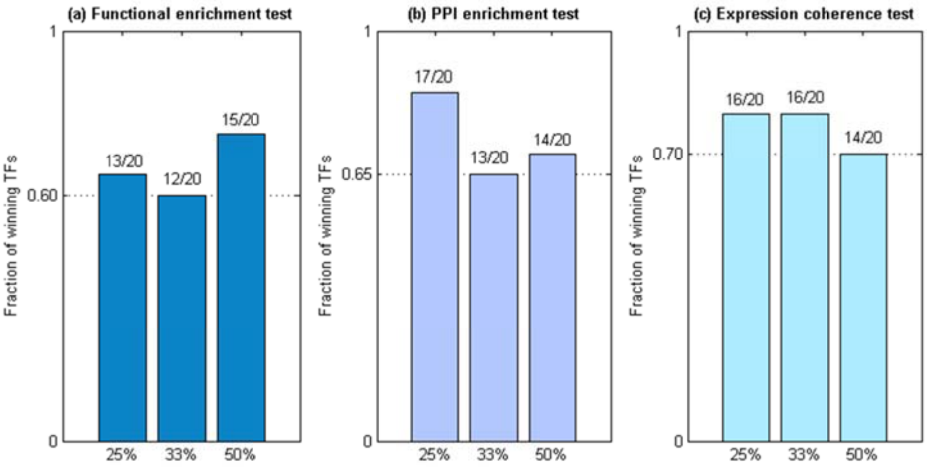

Supplementary Figure 3. The outcomes of the three tests for the 20 TFs under study from SwissRegulon database.

Re(A,k) and *Or(A,k)* are the sets of genes whose promoters contain the "functional" TFBSs of TF *A*, where functional TFBSs of TF *A* are defined as the top *k*% of TF *A*'s TFBSs in our reordered and the original TFBS datasets, respectively. For each of the 20 TFs under study, the three tests were performed on *Re(A,k)* and *Or(A,k)*, where *k*=25, 33 or 50. The performance comparison results of (a) the functional enrichment test, (b) the PPI enrichment test, and (c) the expression coherence test are summarized. Note that TF *A* is called a winning TF if *Re(A,k)* outperformed *Or(A,k)* in the test and the fraction of winning TFs is defined as the number of winning TFs divided by the total number of TFs under study. It can be seen that the fraction of winning TFs is always greater than (a) 0.6 for the functional enrichment test, (b) 0.65 for the PPI enrichment test, and (c) 0.7 for the expression coherence test in all different scenarios, justifying the effectiveness of our post-processor in extracting functional TFBSs from the original TFBS dataset.
