# Supplementary material for: Identifying Functional Transcription Factor Binding Sites in Yeast by Considering Their Positional Preference in the Promoters
Source: PLoS One. 2013 Dec 26;8(12):e83791. doi: 10.1371/journal.pone.0083791 (PMC3873331; doi:10.1371/journal.pone.0083791)
Supplement: Material S6 — Supplementary material 6 provides the ROC analysis results of the TFBS datasets of 30 TFs retrieved from MacIsaac et al.'s study and the TFBS datasets of 20 TFs retrieved from SwissRegulon database. (PDF) [file pone.0083791.s006.pdf]

Note that the original TFBS dataset of a TF is a ranked list of predicted TFBSs of that TF (sorted by the scores generated by an existing TFBS prediction algorithm), where top ranked TFBSs are statistically significant ones. After applying the proposed functional propensity calculator (FPC), the TFBSs in the original TFBS dataset could be reordered, where top ranked TFBSs are now the ones with high functional propensities. That is, the functionality of the FPC is only to reorder the TFBSs in the original TFBS dataset according to the calculated functional propensity of each TFBS. Therefore, the FPC is not a classifier. No training or testing data are used when applying the FPC. The only data used are the original TFBS dataset.

In order to check whether the reordered TFBS dataset (after applying the proposed FPC) outperforms the original TFBS dataset in terms of the true positive rate and the false positive rate, the ROC (receiver operating characteristic) analysis was conducted. Here we use the TFBS dataset of Gcn4 (retrieved from SwissRegulon database [1]) as an example to illustrate the procedure of the ROC analysis. The total number of TFBSs in the retrieved TFBS dataset of Gcn4 is 1085. The procedure of the ROC analysis is as follows. First, divide the original TFBS dataset of Gcn4 into positive samples and negative samples. The positive samples are the TFBSs (in the original TFBS dataset of Gcn4) which are located in the promoters of the genes that are known to be regulated by Gcn4 using the literature evidence [2]. The total number of the positive samples is 472 (i.e. P=472). The negative samples are the TFBSs (in the original TFBS dataset of Gcn4) which are not positive samples. The total number of the negative samples is 613 (i.e. N=613). Second, regard the top k (k=0, 1, 2, …, 1085) TFBSs in the reordered (or the original) dataset as the positive results and the rest as the negative results. Then the true positive rate (TPR) defines how many correct positive results occur among all positive samples (i.e. TPR=TP/P). The false positive rate (FPR) defines how many incorrect positive results occur among all negative samples (i.e. FPR=FP/N). For example, regard the top 300 TFBSs in the reordered (or the original) dataset as the positive results and the rest as the negative results. Then for the reordered dataset, its TPR equals 0.4280 (=202/472) and FPR equals 0.1599 (=98/613), shown as a red box in Figure 1. For the original dataset, its TPR equals 0.3284 (=155/472) and FPR equals 0.2365 (=145/613), shown as a yellow box in Figure 1.

(1)

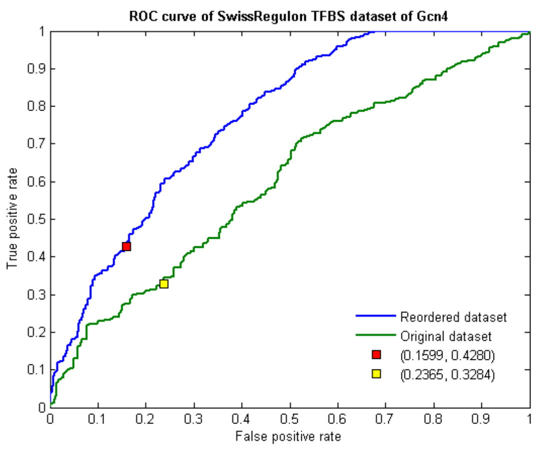

	Rank of reordered dataset	TFBS	Chr	TFBS left end	TFBS right end	FPscore	Positive/Negative samples	Rank of original dataset	TFBS	Chr	TFBS left end	TFBS right end	WMscore	Positive/Negative samples
Positive results	1	GCN4-754	chrXI	18530	18538	8.1832	P	1	GCN4-883	chrXIII	396523	396531	10.3859	P
	2	GCN4-755	chrXI	21113	21121	6.5283	P	2	GCN4-532	chrVIII	141567	141575	10.3859	P
	3	GCN4-886	chrXIII	432392	432400	6.3923	P	3	GCN4-304	chrV	258233	258241	10.3859	P
	4	GCN4-887	chrXIII	432392	432400	6.3923	P	4	GCN4-286	chrV	33814	33822	10.3859	P
	---	---	---	---	---	---	---	---	---	---	---	---	---	---
	300	GCN4-374	chrVI	245427	245435	0.9674	P	300	GCN4-1016	chrXV	207323	207331	7.0941	P
Negative results	---	---	---	---	---	---	---	---	---	---	---	---	---	---
	1082	GCN4-1082	chrXV	762295	762303	-24.8317	N	1082	GCN4-659	chrX	146115	146123	0.3633	P
	1083	GCN4-343	chrV	540521	540529	-25.0962	N	1083	GCN4-50	chrII	378655	378663	-1.5344	P
	1084	GCN4-648	chrX	278006	278014	-25.1867	N	1084	GCN4-948	chrXIV	491311	491319	-2.0364	N
	1085	GCN4-1203	chrIII	128395	128403	-25.1867	N	1085	GCN4-1143	chrXVI	191351	191359	-2.6223	P

The ROC analysis was applied to MacIsaac et al.’s TFBS datasets of 30 TFs (predicted by PhyloCon and Converge algorithms [3]) and the SwissRegulon’s TFBS datasets of 20 TFs (predicted by MotEvo algorithm [1]). Taking the TFBS dataset of Gcn4 as an example, the ROC analysis result (Figure 2a) shows that no matter which TFBS prediction algorithm is used, the AUC (area under curve) of the reordered dataset is larger than that of the original dataset. Actually, in almost all of the TFs under study, our reordered dataset has a larger AUC than the original dataset does and this result is robust against different TFBS prediction algorithms used (Figure 2b), justifying the effectiveness of our post-processor in extracting functional TFBSs from the original TFBS dataset.

(2a)

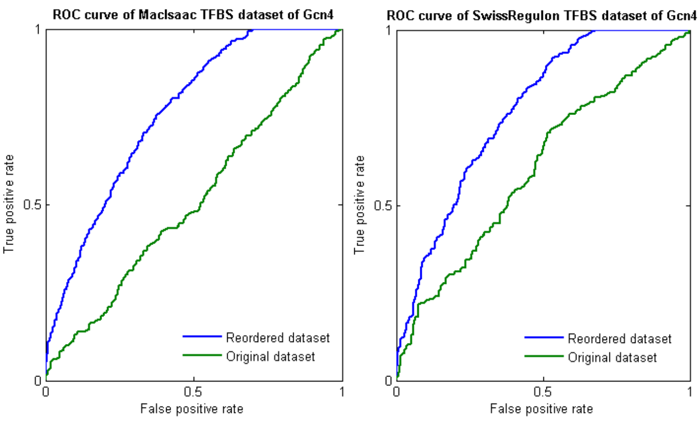

(2b)

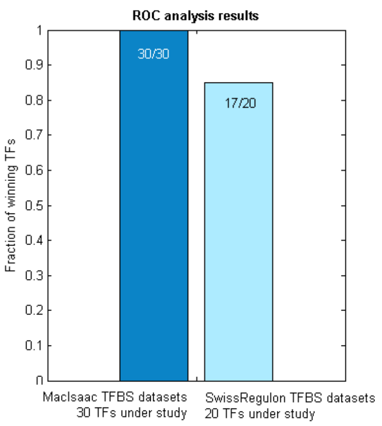

References

1. Pachkov M, Balwierz PJ, Arnold P, Ozonov E, van Nimwegen E (2013) SwissRegulon, a database of genome-wide annotations of regulatory sites: recent updates. Nucleic Acids Res 41: D214-D220.
2. Abdulrehman D, Monteiro PT, Teixeira MC, Mira NP, Lourenço AB, et al. (2011) YEASTRACT: providing a programmatic access to curated transcriptional regulatory associations in Saccharomyces cerevisiae through a web services interface. Nucleic Acids Res 39: D136-D140.
3. MacIsaac KD, Wang T, Gordon DB, Gifford DK, Stormo G, et al. (2006) An improved map of conserved regulatory sites for saccharomyces cerevisiae. BMC Bioinformatics 7: 113.

ROC analysis results of MacIsaac et al.'s TFBS datasets of 30 TFs

It can be seen that Reordered dataset (after applying the FPC) has a larger AUC (area under curve) than Original dataset does in 30 (colored blue) out of the 30 TFs.

No.	TFBS Name	AUC of Reordered dataset	AUC of Original dataset
1	ABF1	0.6658	0.5003
2	ADR1	0.7728	0.6209
3	ARR1	0.6807	0.5081
4	CIN5	0.7920	0.4966
5	CST6	0.7747	0.5029
6	FHL1	0.7866	0.5370
7	FKH1	0.8202	0.5046
8	GCN4	0.7608	0.5096
9	GCR2	0.7277	0.4970
10	GLN3	0.7303	0.4820
11	HAP2	0.8500	0.5253
12	MBP1	0.7743	0.4992
13	MSN2	0.6756	0.4819
14	MSN4	0.6978	0.4923
15	PHD1	0.6692	0.5153
16	PHO2	0.7383	0.5535
17	RAP1	0.7316	0.5171
18	REB1	0.6798	0.4926
19	SFP1	0.7057	0.5035

**20**	**SKN7**	0.6974	0.5405
**21**	**SKO1**	0.7671	0.4972
**22**	**SOK2**	0.6270	0.5034
**23**	**STE12**	0.6259	0.5104
**24**	**SWI4**	0.7181	0.4935
**25**	**SWI5**	0.7656	0.5006
**26**	**SWI6**	0.8205	0.5118
**27**	**TEC1**	0.7428	0.4937
**28**	**YAP1**	0.7771	0.4945
**29**	**YAP5**	0.6998	0.5073
**30**	**YAP6**	0.7557	0.4720

ROC analysis results of SwissRegulon's TFBS datasets of 20 TFs

It can be seen that Reordered dataset (after applying the FPC) has a larger AUC (area under curve) than Original dataset does in 17 (colored blue) out of the 20 TFs.

**No.**	**TFBS Name**	**AUC of Reordered dataset**	**AUC of Original dataset**
**1**	**ABF1**	0.6935	0.6660
2	CBF1	0.7613	0.8169
**3**	**CIN5**	0.7976	0.6298
**4**	**CRZ1**	0.7652	0.5562
**5**	**FHL1**	0.7169	0.5413
**6**	**GCN4**	0.7669	0.6022
**7**	**MBP1**	0.7295	0.6365
**8**	**MCM1**	0.7379	0.7145
**9**	**MSN2**	0.6903	0.5172
**10**	**MSN4**	0.7394	0.5398
**11**	**PDR1**	0.7147	0.5720
**12**	**PHD1**	0.7565	0.5116
13	RAP1	0.7123	0.7281
14	REB1	0.6660	0.7728
**15**	**SFP1**	0.6493	0.5825
**16**	**SOK2**	0.7281	0.5273

**17**	**STE12**	0.6981	0.6196
**18**	**SWI4**	0.7362	0.5969
**19**	**TEC1**	0.7879	0.5982
**20**	**YAP1**	0.7260	0.5211

ROC curves of each TF under study

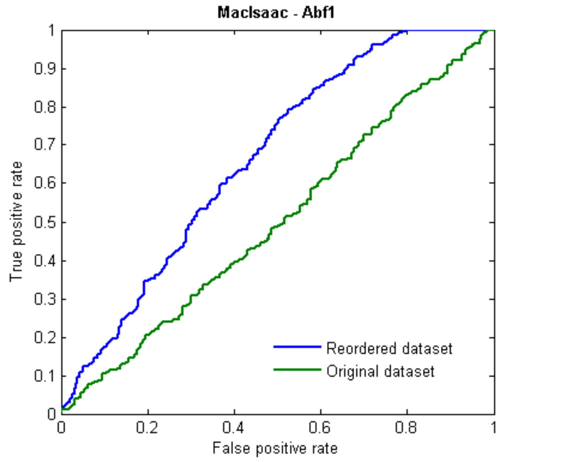

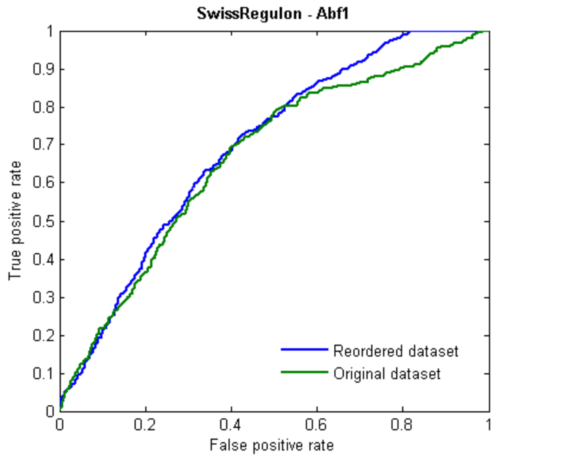

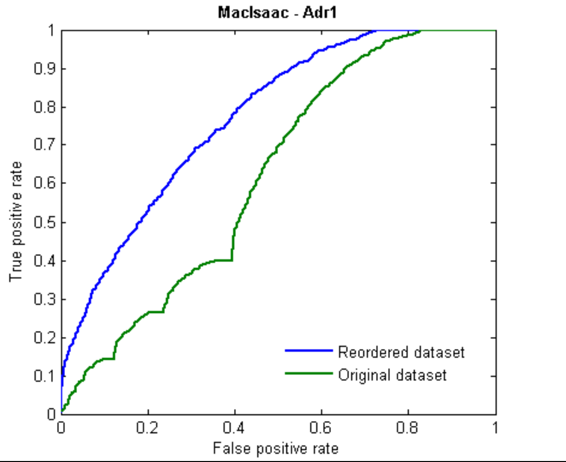

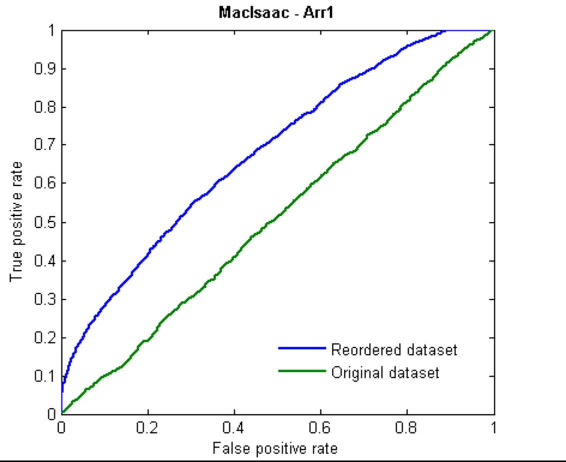

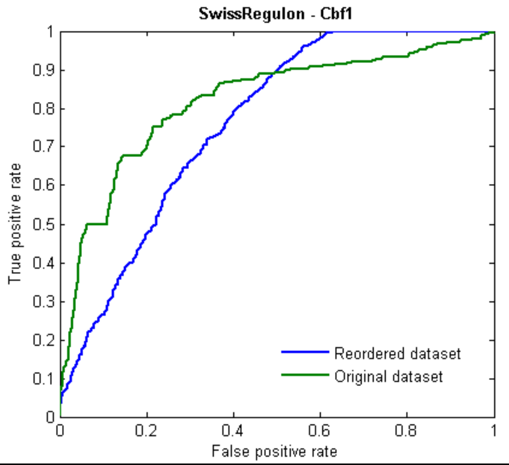
SwissRegulon - Cbf1
True positive rate
False positive rate
Reordered dataset
Original dataset

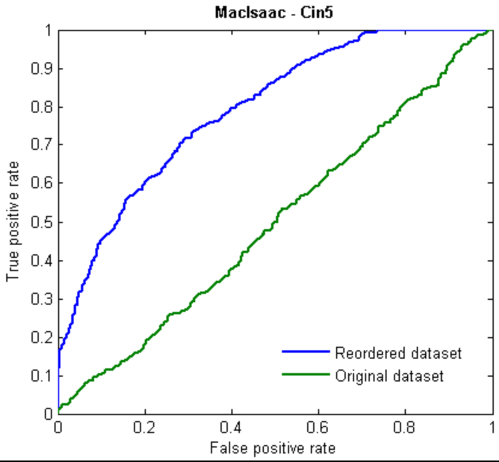
MacIsaac - Cin5
True positive rate
False positive rate
Reordered dataset
Original dataset

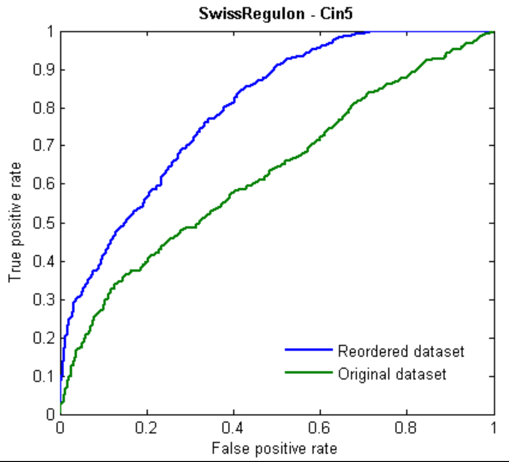
SwissRegulon - Cin5
True positive rate
False positive rate
Reordered dataset
Original dataset

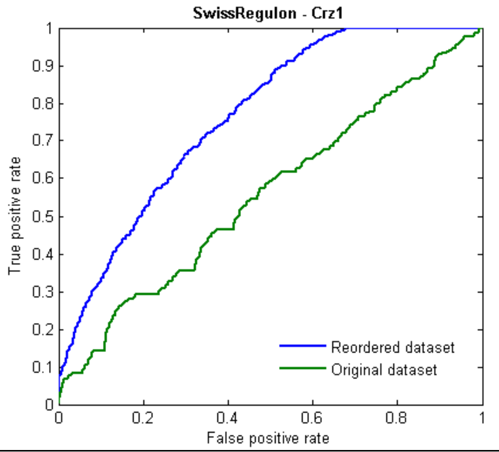
SwissRegulon - Crz1
True positive rate
False positive rate
Reordered dataset
Original dataset

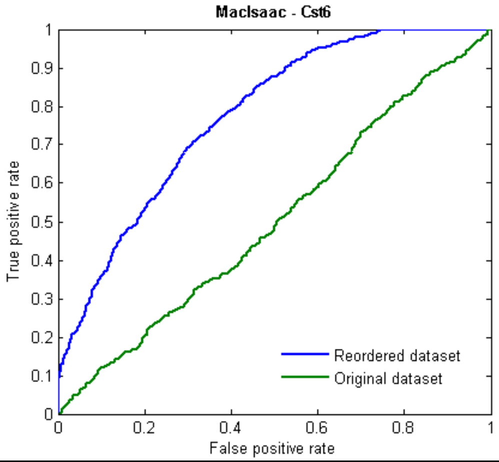
MacIsaac - Cst6
True positive rate
False positive rate
Reordered dataset
Original dataset

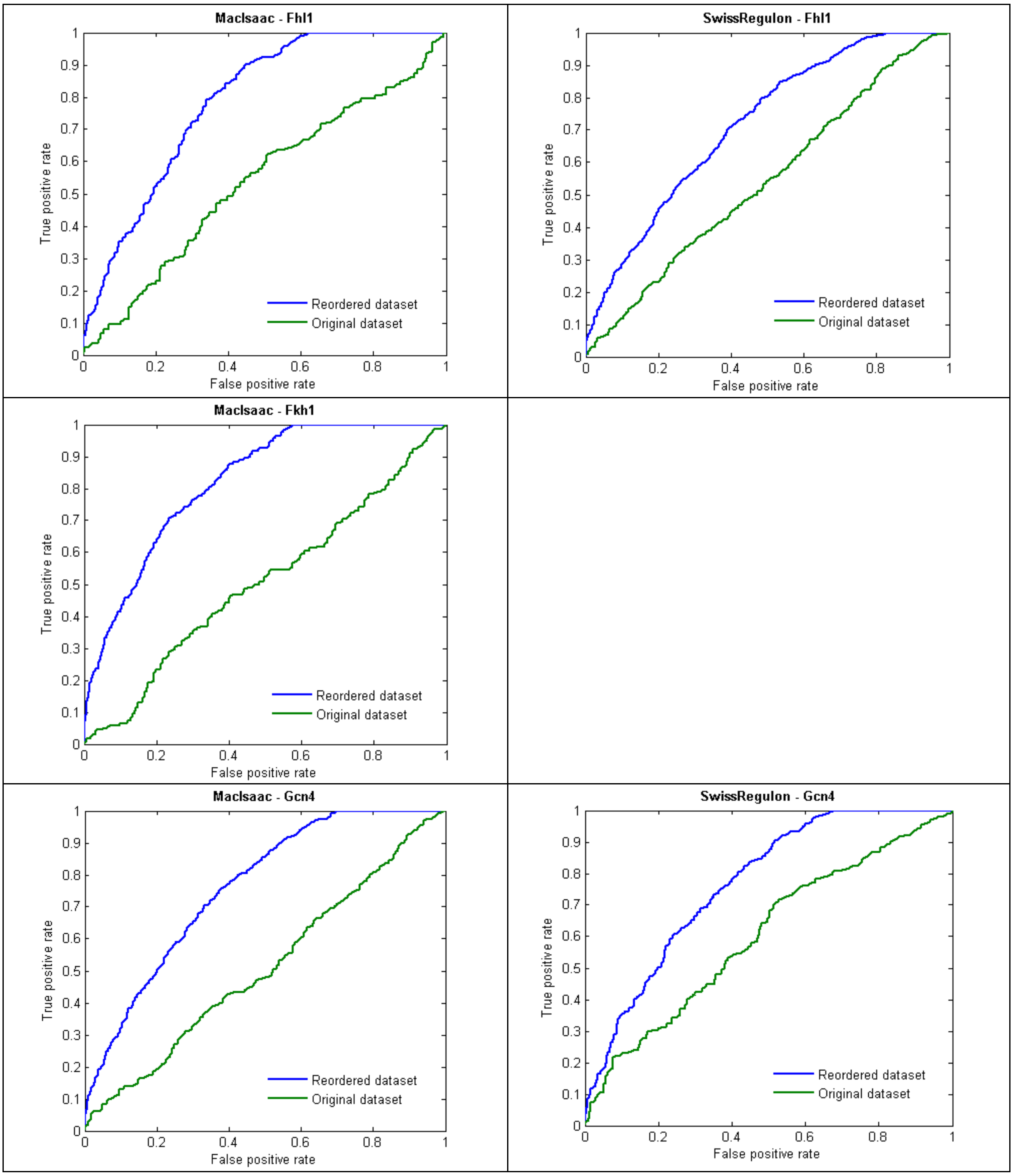
MacIsaac - Fhl1
True positive rate
False positive rate
Reordered dataset
Original dataset
SwissRegulon - Fhl1
True positive rate
False positive rate
Reordered dataset
Original dataset
MacIsaac - Fkh1
True positive rate
False positive rate
Reordered dataset
Original dataset
MacIsaac - Gcn4
True positive rate
False positive rate
Reordered dataset
Original dataset
SwissRegulon - Gcn4
True positive rate
False positive rate
Reordered dataset
Original dataset

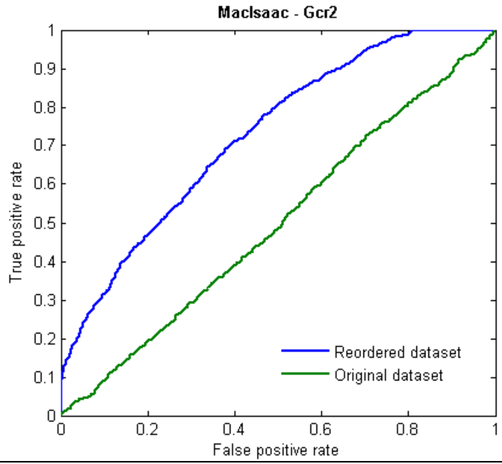
MacIsaac - Gcr2
True positive rate
False positive rate
Reordered dataset
Original dataset
0
0.1
0.2
0.3
0.4
0.5
0.6
0.7
0.8
0.9
1
0
0.2
0.4
0.6
0.8
1

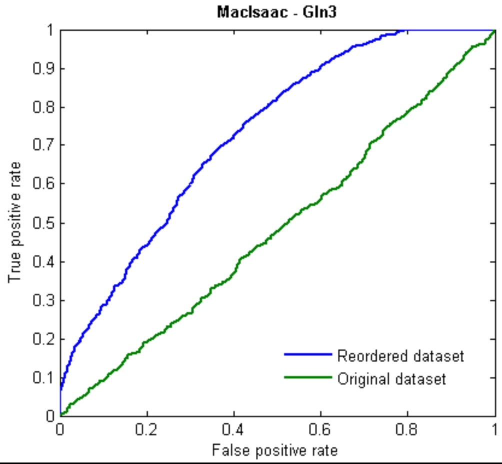
MacIsaac - Gln3
True positive rate
False positive rate
Reordered dataset
Original dataset
0
0.1
0.2
0.3
0.4
0.5
0.6
0.7
0.8
0.9
1
0
0.2
0.4
0.6
0.8
1

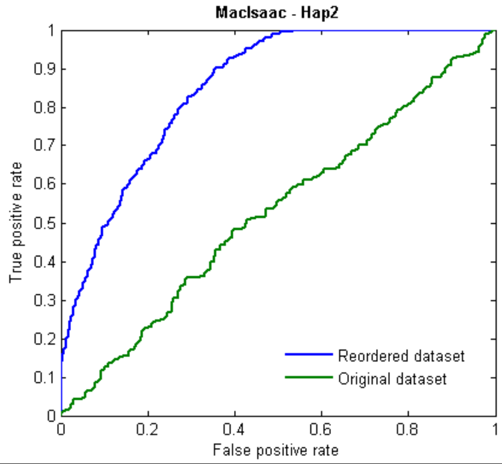
MacIsaac - Hap2
True positive rate
False positive rate
Reordered dataset
Original dataset
0
0.1
0.2
0.3
0.4
0.5
0.6
0.7
0.8
0.9
1
0
0.2
0.4
0.6
0.8
1

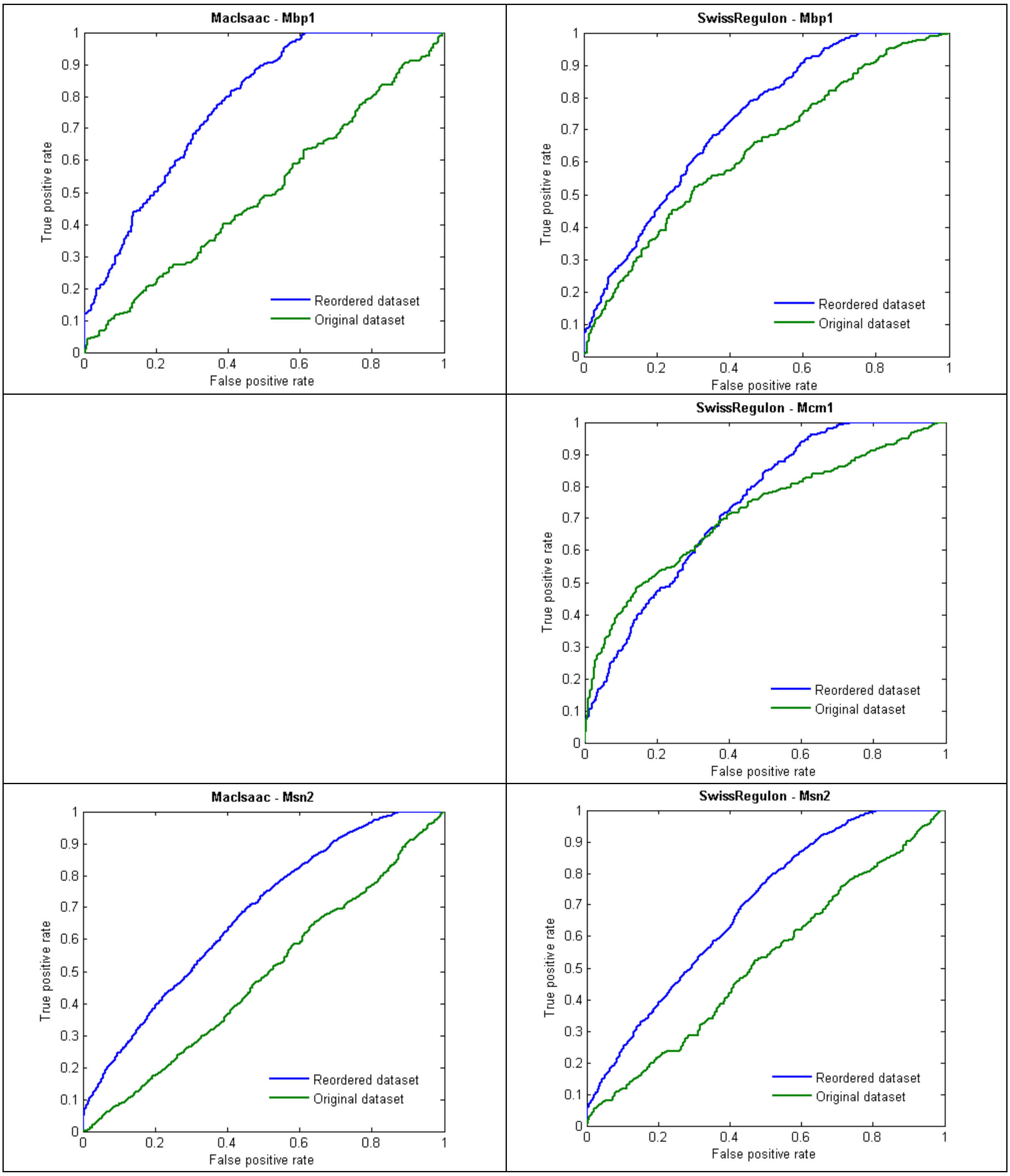
MacIsaac - Mbp1
SwissRegulon - Mbp1
SwissRegulon - Mcm1
MacIsaac - Msn2
SwissRegulon - Msn2
True positive rate
False positive rate
Reordered dataset
Original dataset
0
0.1
0.2
0.3
0.4
0.5
0.6
0.7
0.8
0.9
1

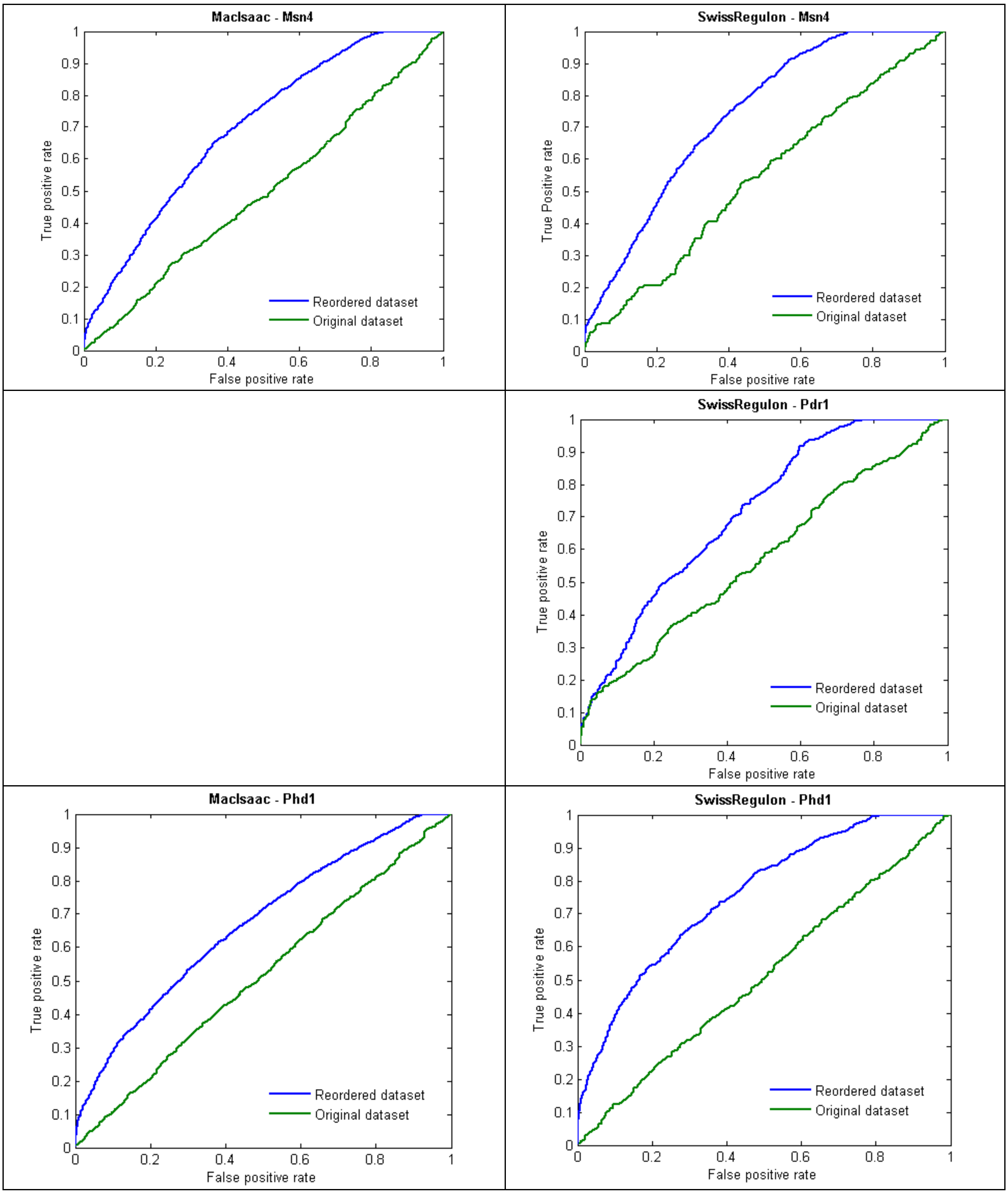
MacIsaac - Msn4
SwissRegulon - Msn4
SwissRegulon - Pdr1
MacIsaac - Phd1
SwissRegulon - Phd1
True positive rate
True Positive rate
False positive rate
Reordered dataset
Original dataset
0
0.1
0.2
0.3
0.4
0.5
0.6
0.7
0.8
0.9
1

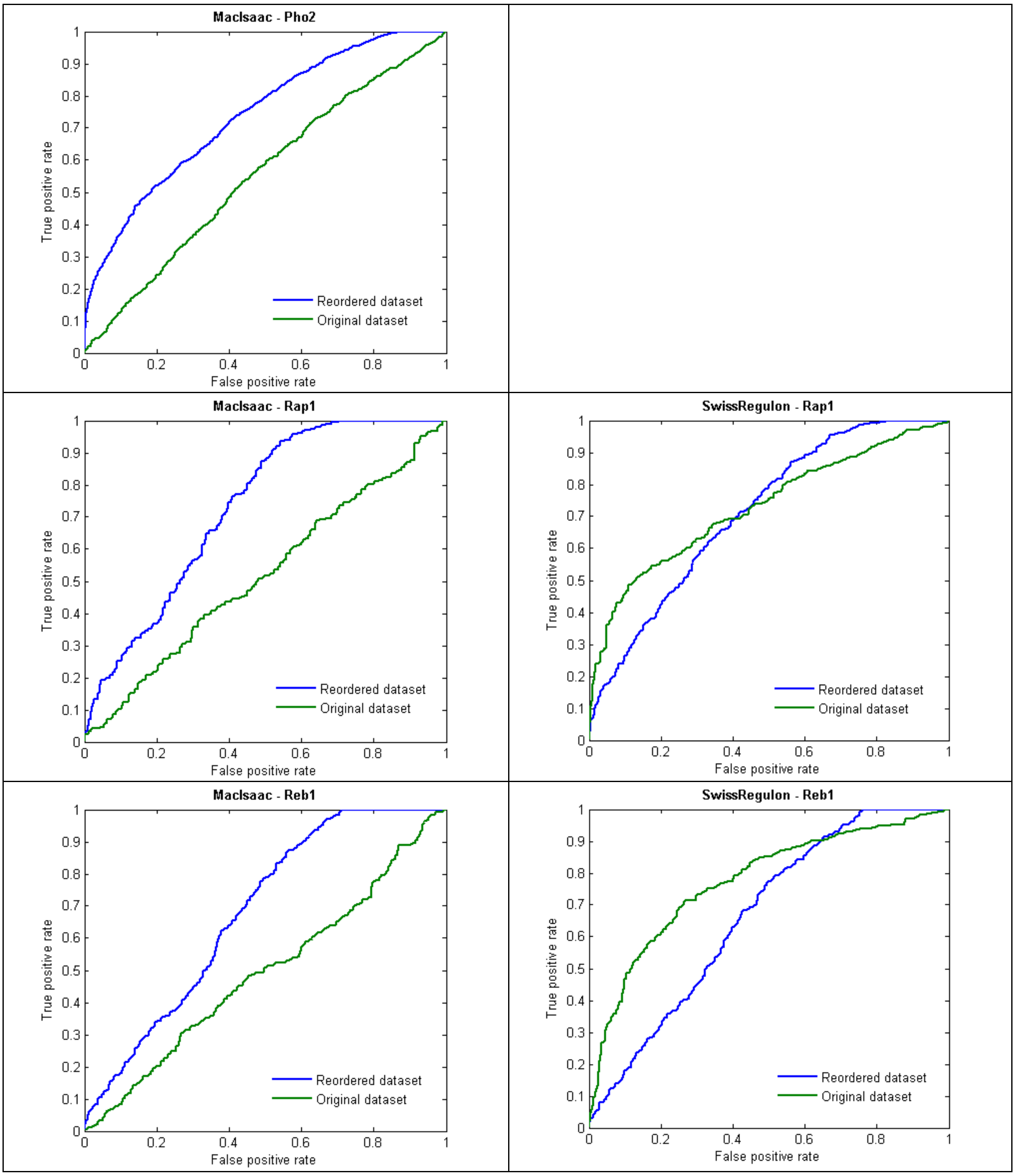
MacIsaac - Pho2
True positive rate
False positive rate
Reordered dataset
Original dataset
MacIsaac - Rap1
True positive rate
False positive rate
Reordered dataset
Original dataset
SwissRegulon - Rap1
True positive rate
False positive rate
Reordered dataset
Original dataset
MacIsaac - Reb1
True positive rate
False positive rate
Reordered dataset
Original dataset
SwissRegulon - Reb1
True positive rate
False positive rate
Reordered dataset
Original dataset

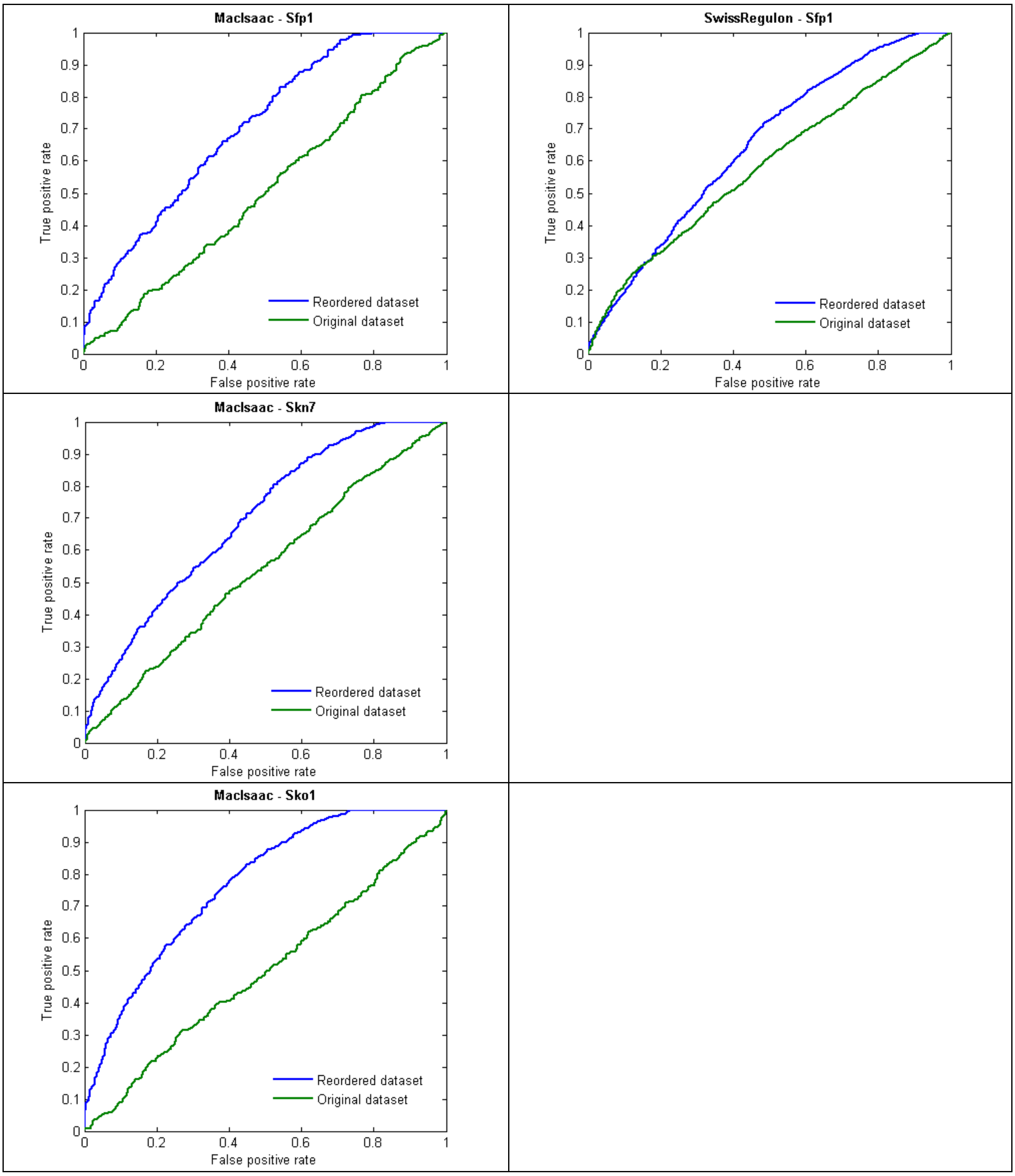
MacIsaac - Sfp1
True positive rate
False positive rate
Reordered dataset
Original dataset
SwissRegulon - Sfp1
True positive rate
False positive rate
Reordered dataset
Original dataset
MacIsaac - Skn7
True positive rate
False positive rate
Reordered dataset
Original dataset
MacIsaac - Sko1
True positive rate
False positive rate
Reordered dataset
Original dataset

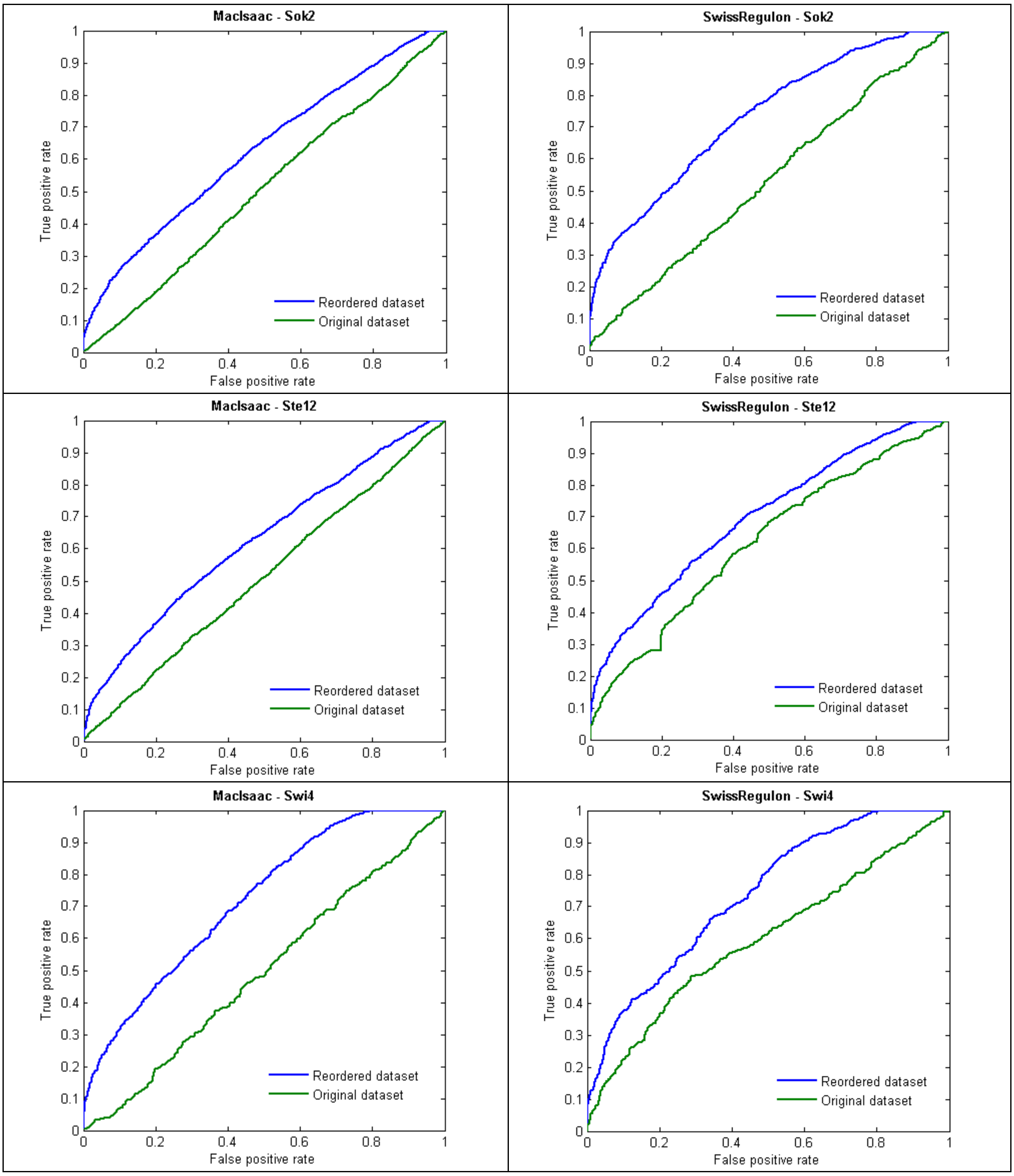
MacIsaac - Sok2
SwissRegulon - Sok2
MacIsaac - Ste12
SwissRegulon - Ste12
MacIsaac - Swi4
SwissRegulon - Swi4
True positive rate
False positive rate
Reordered dataset
Original dataset
0
0.1
0.2
0.3
0.4
0.5
0.6
0.7
0.8
0.9
1

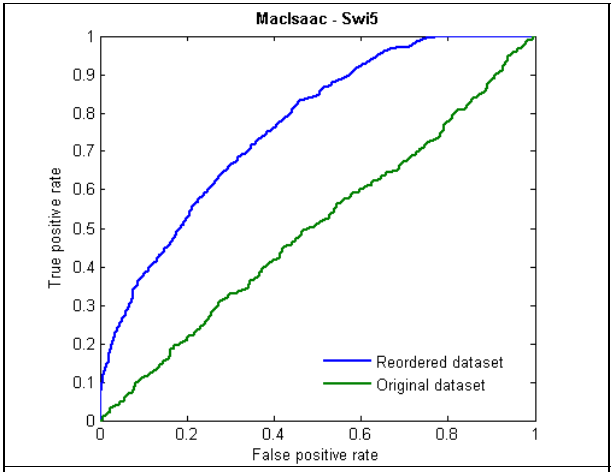
MacIsaac - Swi5
True positive rate
False positive rate
Reordered dataset
Original dataset

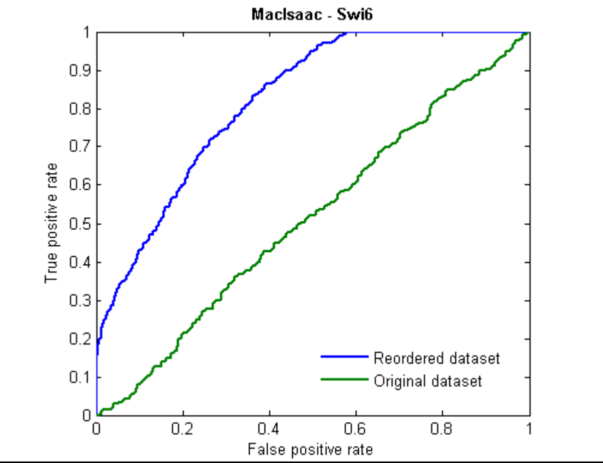
MacIsaac - Swi6
True positive rate
False positive rate
Reordered dataset
Original dataset

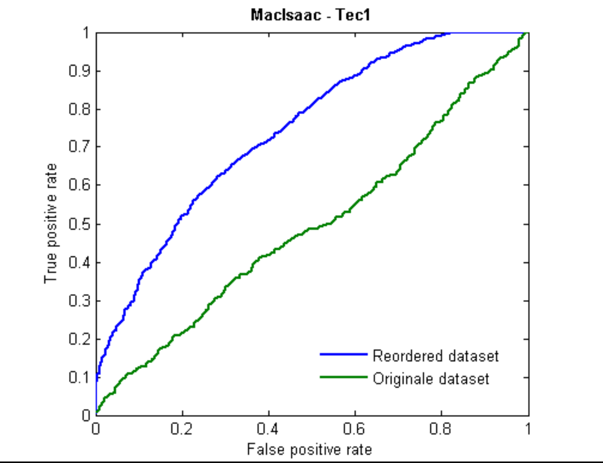
MacIsaac - Tec1
True positive rate
False positive rate
Reordered dataset
Originale dataset

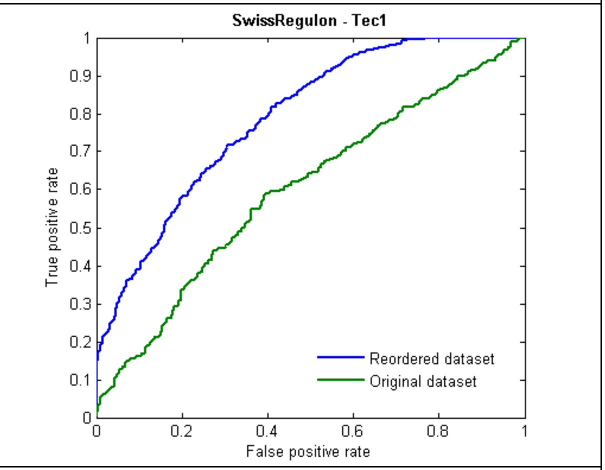
SwissRegulon - Tec1
True positive rate
False positive rate
Reordered dataset
Original dataset

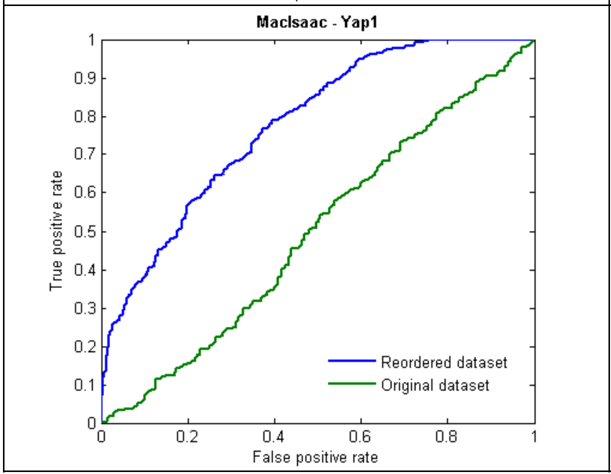
MacIsaac - Yap1
True positive rate
False positive rate
Reordered dataset
Original dataset

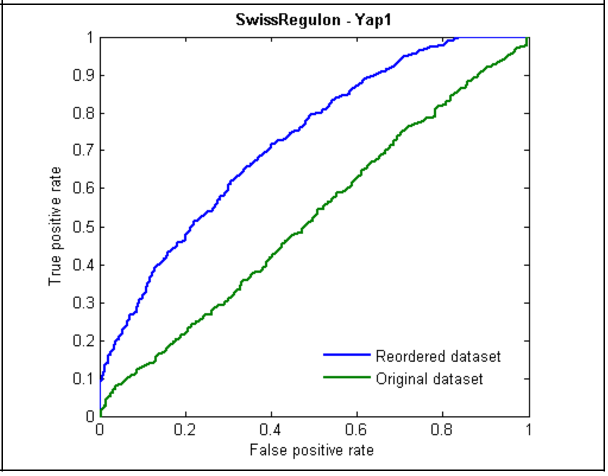
SwissRegulon - Yap1
True positive rate
False positive rate
Reordered dataset
Original dataset

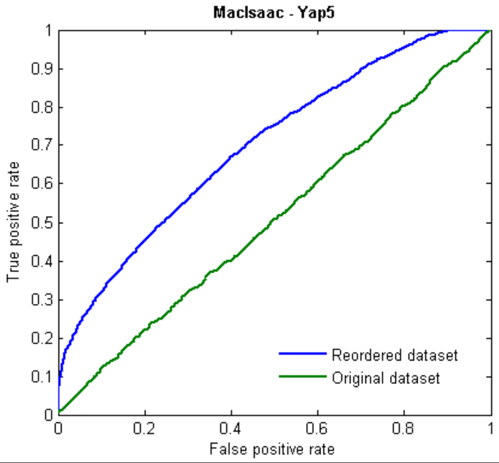
MacIsaac - Yap5
True positive rate
False positive rate
Reordered dataset
Original dataset

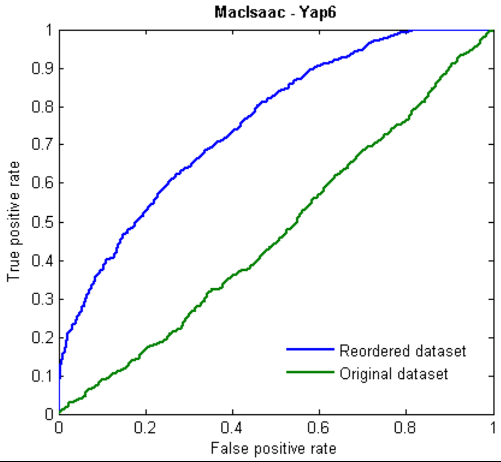
MacIsaac - Yap6
True positive rate
False positive rate
Reordered dataset
Original dataset
